# Assessment of Risk Factors Associated with Cardiovascular Diseases in Overweight Women

**DOI:** 10.3390/nu13103658

**Published:** 2021-10-19

**Authors:** María-José Castro, José-María Jiménez, María López, María-José Cao, Manuel Durán, Irene Albertos, Sara García, Jaime Ruiz-Tovar

**Affiliations:** 1 Nursing Faculty, University of Valladolid, 47005 Valladolid, Spain; mariajose.castro@uva.es (M.-J.C.); maria.lopez.vallecillo@uva.es (M.L.); mjcao@enf.uva.es (M.-J.C.); irenealbertos@uva.es (I.A.); sara.garcia.villanueva@uva.es (S.G.); 2 Department of Surgery, Rey Juan Carlos Hospital, 28933 Madrid, Spain; manuel.duran@hospitalreyjuancarlos.es; 3 Department of Surgery, University Alfonso X, 28691 Madrid, Spain; jruiztovar@gmail.com

**Keywords:** mammary volume, body mass index, cardiovascular risk, overweight, women

## Abstract

The assessment of anthropometric variables has been shown to be useful as a predictor of cardiovascular risk in overweight and obese patients. The aim of this study was to determine the usefulness of the relationship between breast volume and body mass index as an indicator of cardiovascular risk in premenopausal women with overweight and mild obesity. A prospective observational study of 93 premenopausal women was performed. Evaluation of anthropometric measures included age, body mass index, waist and hip circumferences, breast projection, and ptosis. Cardiovascular risk factors were evaluated using the Framingham cardiovascular risk score, the triglycerides/HDL cholesterol ratio and the waist-hip ratio. Ninety-three women were included, with a mean 36.4 ± 7.5 years. Mean BMI was 27.3 ± 1.9 kg/m^2^, waist-to-Hip ratio was 0.8 ± 0.07, and mammary volume was 1045 ± 657.4 cm^3^. Mean body fat mass was 30.6 + 3.6% and mean visceral fat was 6.6 + 3.2%. The mean triglycerides to HDL ratio was 1.7 ± 0.8 and waist-to-hip ratio was 0.8 ± 0.07. Breast volume related to body mass index can be used as a predictor of cardiovascular risk in premenopausal women who are overweight and mildly obese.

## 1. Introduction

At present, obesity is a life-threatening condition in developed countries. Thus, it is considered as a modern global epidemic disease [1]. In Western countries, the prevalence of overweight and obesity reaches 38% and 14% of the total population, revealing that over 50% of the population presents an excess of weight. In the last decade, this prevalence has shown a significant increase and there is a trend towards a progressive rise in the coming years, based mostly on the high prevalence of obesity and overweight among children and adolescents. It has been estimated that in 2030, obesity rates could reach up to 20%, and the addition of obesity and overweight could be around 60% [2,3].

Obesity has been associated with a broad spectrum of diseases, including type 2 diabetes mellitus, hypertension, dyslipidemia, sleep apnea/hypopnea syndrome, non-alcoholic fatty liver disease and even diverse neoplasms, among others [1]. Altogether, they imply an increase in morbidity and mortality, and a reduction in quality of life. Cardiovascular disease and diabetes are the leading causes of death related to high body mass index (BMI) [4].

Different multivariable models are used to calculate the risk of suffering a cardiovascular disease (CVD). The Framingham Risk Score (FRS) is the most widely used in clinical practice. It determines the risk of suffering a first event of coronary heart disease, cerebrovascular disease, peripheral artery disease and heart failure in the next 10 years. The FRS is calculated with an algorithm including age, gender, total cholesterol, systolic blood pressure, smoking status and the presence of type 2 diabetes [5]. Other CVD risk scores include different analytical values, such as the ratio of triglycerides to high-density lipoprotein–cholesterol (HDL–cholesterol), which has shown to be a strong predictor of myocardial infarction, especially when values surpass 4.5 [6,7]. Finally, several anthropometric parameters, such as waist-to-hip ratio [8] or waist-to-height ratio [9], have demonstrated a certain predictive effect on the CVD risk.

A recent study by our group has established the Mammary Volume-to-Body Mass Index (MV-BMI) ratio as a predictive factor for CVD risk in morbidly obese women, as it has an inverse correlation with the FRS and triglycerides/HDL–cholesterol ratio [8], determining that those women with an MV-BMI over 60 (cm^3^/(kg/m^2^) had significantly lower values of FRS and triglycerides-to-HDL ratios and lower T2D, hypertension and dyslipidemia rates, without significant differences in BMI.

Anthropometric parameters are useful in clinical practice to assess CVD risk, as they can be easily applied, without the need of complex devices or analytical data to be included in formulas. The waist-to-hip ratio is clinically useful, but a more accurate index must still be explored.

Women’s breasts are composed of the mammary gland and connective/adipose tissue. Because these tissues have hormone receptors, their sizes and volumes fluctuate according to hormonal changes. The adipose component increases with obesity. However, excess fat distribution is not homogeneous among different women. Some obese women develop big breasts with weight increases, whereas other subjects show a different distribution of adiposity and do not significantly increase the size of their breasts [10,11]. The aim of this study was to determine the usefulness of the MV-BMI ratio as an indicator of CVD risk in overweight and mildly obese premenopausal women.

## 2. Materials and Methods

A prospective observational study of 93 consecutive premenopausal women, attending a nutritional outpatient clinic and complaining of excess weight, was performed. Inclusion criteria were patients with body mass index (BMI) between 25 and 35 kg/m^2^. Exclusion criteria included all kinds of previous breast surgeries.

### 2.1. Definitions

**Figure 1:**
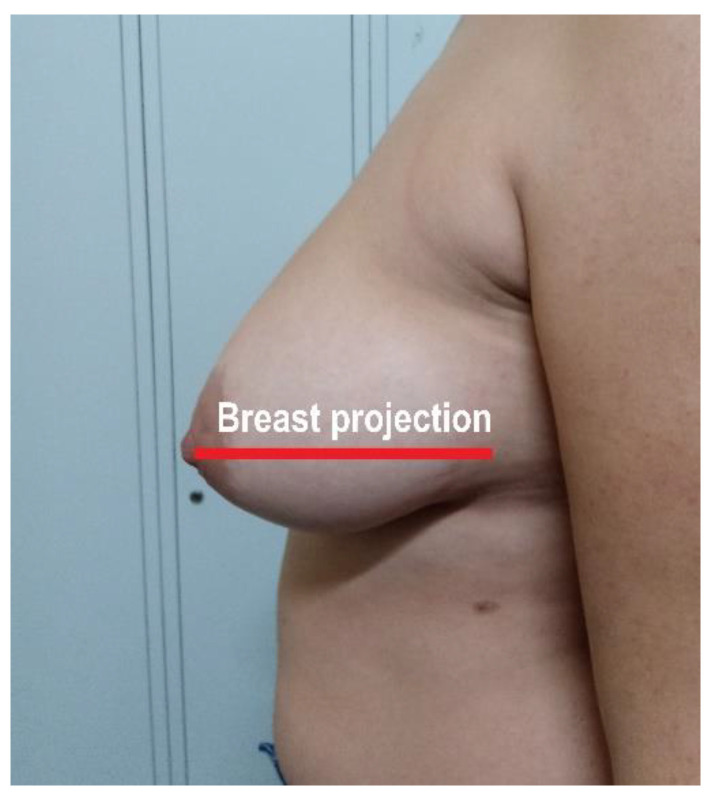
Illustration of breast projection.

Mammary ptosis was defined as the clavicle–nipple length. Projection was measured as the distance between the points of implantation of the breast in the chest up to the nipple in a standing patient (Figure 1).

**Figure 2:**
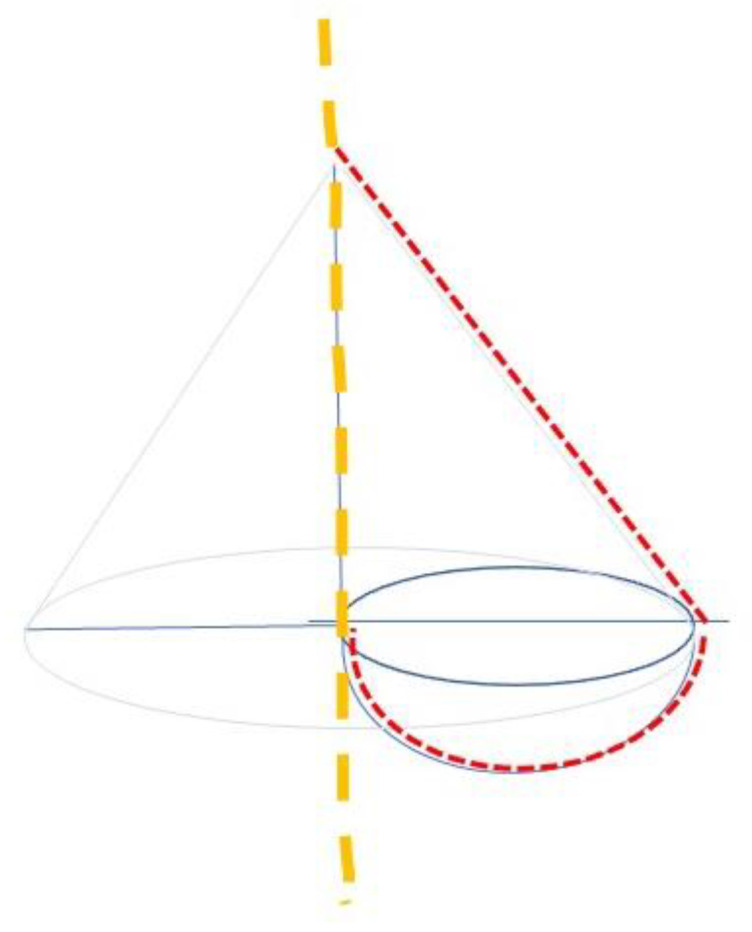
Geometric model for calculating breast volume.

Mammary volume was calculated based on the geometry of the breast model, as described by Copcu [11]. According to this geometric model, the following formula was developed, considering the upper part of the breast as a half cone and the lower part as a half globe (Figure 2).

The volume of the half globe was calculated following the formula:

½ × Pi × r (cm)^3^

r = Projection (cm)/2.

The volume of the half cone was calculated following the formula:

½ × 1/3 × Pi × r (cm)^2^ × height (cm)

height = Projection (cm); r = Ptosis (cm).

Therefore, the complete formula to calculate the total mammary volume was:

(½ × Pi × Projection (cm)^3^) + (½ × 1/3 × Pi × Ptosis (cm)^2^ × Projection (cm))

MV-BMI is then calculated following the formula:

[(½ × Pi × Projection (cm)^3^) + (½ × 1/3 × Pi × Ptosis (cm)^2^ × Projection (cm))]/[Weight (Kg)/Height (m)^2^]

In our previous study we determined a cut-off point of MV-BMI > 60 (cm^3^/(Kg/m^2^), associated with an FRS of <1% in morbidly obese premenopausal females [10].

Mammary measurements were carried out 7 days after menstruation, in order to avoid changes in mammary volume associated with menstruation or ovulation.

### 2.2. Analysis of Body Composition

Body composition measures were evaluated between 8:00 and 10:00 a.m., after at least 8 h of fasting, with an empty bladder. Alcohol consumption and exercise were forbidden 8 h before the test. Bioelectrical impedance analysis (Tanita BC-420MA, Tanita, Tokyo, Japan) was used to assess body composition, as a validated method [12].

### 2.3. Variables

Anthropometric measurements included age, BMI, waist and hip circumferences, mammary projection and ptosis.

Cardiovascular risk factor was assessed by the FRS (FRS) [5]. The triglycerides/HDL–cholesterol ratio and the waist-to-hip ratio were also calculated.

Adiposity and visceral fat were assessed by bioelectrical impedance analysis.

The MV-BMI ratio was investigated as a cardiovascular risk factor and compared with the triglycerides/HDL–cholesterol ratio, the waist-to-hip ratio and adiposity. The results of this ratio are expressed as (cm^3^/(kg/m^2^)).

### 2.4. Statistical Analysis

Statistical analysis was performed using IBM SPSS v. 22.0 software (IBM, Armonk, NY, USA). Quantitative variables were defined by mean and standard deviation (median and range in non-Gaussian variables). Qualitative variables were defined by number of cases and percentages.

Correlation between quantitative variables was performed with Pearson and Spearman correlation tests. Paired Student *t* tests were used to compare data before and after surgery.

Values of *p* < 0.05 were considered significant. Receiver operating characteristic (ROC) curve analysis was performed, and the respective areas under the curve (AUC) were calculated to evaluate predictive values for the investigated analytical values. Cut-off points were investigated. The sensitivity and specificity of these parameters were then calculated.

## 3. Results

**Figure 3:**
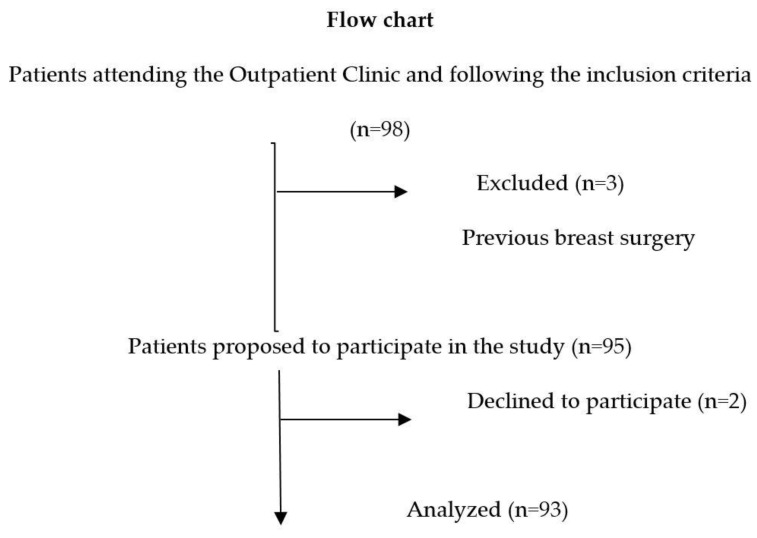
Flow chart of the patients analyzed. Ninety-three premenopausal women were initially included, with a mean age of 36.4 ± 7.5 years and a mean BMI of 27.3 ± 1.9 kg/m^2^. Anthropometric measurements are described in Table 1.

A flow chart of the patients analyzed is shown in Figure 3.

### 3.1. Bioimpedance Analysis

Mean body fat mass was 30.6 + 3.6% and mean visceral fat was 6.6 + 3.2%.

### 3.2. Cardiovascular Risk Factors Assessment

The mean cardiovascular risk in the next 10 years following the FRS was 3.8 ± 3%. The mean triglycerides to HDL ratio was 1.7 ± 0.8. The mean waist-to-hip ratio was 0.8 ± 0.07.

### 3.3. Correlation between MV-BMI and Cardiovascular Risk Factors

MV-BMI showed an inverse correlation with the FRS (Spearman—0.670; *p* = 0.034), and a significant inverse correlation with the triglycerides/HDL–cholesterol ratio (Spearman—0.675; *p* = 0.016).

Correlating it with body composition, MV-BMI presented an inverse correlation with body fat mass (Spearman—0.825; *p* = 0.001) and a significant inverse correlation with visceral fat mass (Spearman—0.628; *p* = 0.001).

Referring to anthropometric parameters, MV-BMI did not reach a significant correlation with the waist-to-hip ratio (*p* = 0.738).

### 3.4. Evaluation of MV-BMI or Waist-To-Hip Ratio as Best Anthropometric Predictor of FRS

As previously mentioned, MV-BMI showed an inverse correlation with the FRS (Spearman—0.670; *p* = 0.034). Waist-to-hip ratio did not show a significant correlation with the FRS (*p* = 0.618). Consequently, MV-BMI was a better predictor of cardiovascular risk than waist-to-hip ratio.

## 4. Discussion

Women’s breasts are composed of the mammary gland and connective/adipose tissue. Because these tissues have hormone receptors, their size and volume fluctuate according to hormonal changes. The adipose component increases with obesity. However, excess fat distribution is not homogeneous among different women. Some obese women develop big breasts with an increase in weight, whereas other subjects show a different distribution of adiposity and do not significantly increase the size of their breasts [13,14].

The female sex hormones (principally estrogens) in conjunction with growth hormone promote the sprouting, growth, and development of the breasts. At menopause, breast atrophy occurs, coinciding the decrease in size with the decline in the levels of circulating estrogen [15]. Several studies have hypothesized that the volume of the mammary glandular tissue in women is associated with estrogen levels, whereas BMI correlates with higher percentages of fat in the breast [16,17].

It is widely known that premenopausal estrogen release acts as a cardiovascular protective factor, while after the menopause, this estrogen decrease elevates the CVD risk up to similar levels to males [18]. As estrogen segregation differs in the diverse stages of the menstrual cycle, it is difficult to establish a total amount of this hormone, but altogether, it may have a trophic effect on the development of mammary glandular tissue. The effect of estrogenic status can also be reflected in the reduction in CVD risk.

Janiszewski et al. [19] reported that breast volume was an independent predictor of visceral fat in premenopausal women, and this can be considered as a CVD risk factor. However, they failed to demonstrate a significant association with the lipid or glycemic profile. In contrast, the MV-BMI ratio in our study was inversely associated with the serum triglyceride levels and with the triglycerides/HDL–cholesterol ratio. Moreover, MV-BMI was also inversely associated with the visceral fat, as assessed by bioelectrical impedance.

According to the results obtained in the present study, it appears that overweight premenopausal women with bigger breasts have a better metabolic profile overall. It is true that a greater breast volume can depend on gland or fatty tissue. With this approach, the composition of the breast remains unknown.

Handheld, three-dimensional scanners with specific software and MRI are considered the most accurate methods for the calculation of mammary volume [20]. However, specific software is not universally available. The formula for mammary volume calculation used in this study is only an approximation. However, the associations determined with different cardiovascular risk indexes made this formula a useful tool to evaluate cardiovascular risk.

This formula has been previously applied to severely obese premenopausal women candidates for bariatric surgery [10], and in the present study, to premenopausal women with overweight or mild obesity. It must be still elucidated if this formula is also applicable for postmenopausal women, as a predictive factor for CVD, as this age range is more prone to developing cardiovascular events. Furthermore, this formula to estimate mammary volume must be validated with gold-standard methods (MRI).

Despite the waist-to-hip ratio being a useful parameter to determine CVD risk, we failed to demonstrate an association between this parameter and the MV-BMI. This was likely due to the small sample size. Future studies with a larger number of subjects included must be conducted to confirm further associations.

One of the main limitations of the routine implementation of MV-BMI as an indicator of CVD can be that this tool requires complex time-consuming calculations and inconvenient measurements for women that can vary with human error. However, in our study, this ratio has demonstrated a higher predictive value of CVD than the waist-to-hip ratio. Further studies must confirm the usefulness of this index.

## 5. Conclusions

MV-BMI showed an inverse correlation with the FRS and triglycerides/HDL–cholesterol ratio, body fat mass and visceral fat mass. MV-BMI can be used as a predictive factor of cardiovascular risk in premenopausal overweight women.

## Figures and Tables

**Table 1:** Anthropometric measurements.

Waist circumference (cm)	80.6 ± 9.6
Hip circumference (cm)	101.7 ± 10.2
Waist-to-hip ratio	0.8 ± 0.07
Mammary projection (cm)	18.8 ± 3.6
Ptosis (cm)	17.1 ± 7.3
Mammary volume (cm^3^)	1045 ± 657.4
Mammary volume/BMI ratio (cm^3^/(kg/m^2^)	38.4 ± 24.3

BMI: body mass index.
